# Thyroid dysfunction in Iranian pregnant women: a systematic review and meta-analysis

**DOI:** 10.1186/s12884-020-03040-5

**Published:** 2020-07-14

**Authors:** Farnaz Sepasi, Tayebeh Rashidian, Mehdi Shokri, Gholamreza Badfar, Fatemeh Kazemi, Milad Azami

**Affiliations:** 1 Department of Obstetrics and Gynecology, School of Medicine, Tabriz University of Medical Sciences, Tabriz, Iran; 2 Department of Obstetrics and Gynecology, School of Medicine, Ilam University of Medical Sciences, Ilam, Iran; 3 Department of Pediatrics, School of Medicine, Ilam University of Medical Sciences, Ilam, Iran; 4 Department of Pediatric, Faculty of Medicine, Ahvaz Jundishapour University of Medical Sciences, Ahvaz, Iran; 5 School of Medicine, Qazvin University of Medical Sciences, Qazvin, Iran; 6 School of Medicine, Ilam University of Medical Sciences, Ilam, Iran

**Keywords:** Thyroid dysfunction, Iran, Pregnant women, Meta-analysis

## Abstract

**Background:**

Thyroid dysfunction during pregnancy is associated with adverse outcomes for both mother and fetus. The present meta-analysis was conducted to evaluate thyroid dysfunction in Iranian pregnant women.

**Methods:**

We registered this review at PROSPERO (registration number: CRD42020166655). The research steps in this systematic review and meta-analysis were performed according to the MOOSE protocol, and finally, reports were provided based on the PRISMA guidelines. The literature search was performed in October 2019 using the international online databases, including Web of Science, Ovid, Science Direct, Scopus, EMBASE, PubMed/Medline, Cochrane Library, EBSCO, CINAHL, Google Scholar as well as national databases were reviewed. Data were extracted after applying the inclusion and exclusion criteria and qualitative evaluation of the studies. I^2^ index and Q test were used to assess differences in studies. All analyses were performed using Comprehensive Meta-Analysis Software. *P*-value less than 0.05 was considered statistically significant. We identified 1261 potential articles from the databases, and 426 articles remained after removing the duplicate and unrelated studies. After evaluating the full text, 52 articles were removed.

**Results:**

Finally, 19 eligible studies including 17,670 pregnant women included for meta-analysis. The prevalence of thyroid dysfunction in Iranian pregnant women was 18.10% (95%CI: 13.89–23.25). The prevalence of hypothyroidism, clinical hypothyroidism, and subclinical hypothyroidism in Iranian pregnant women was respectively estimated to be 13.01% (95%CI: 9.15–18.17), 1.35% (95%CI: 0.97–1.86) and 11.90% (95%CI: 7.40–18.57). The prevalence of hyperthyroidism, clinical hyperthyroidism, and subclinical hyperthyroidism in Iranian pregnant women was respectively estimated to be 3.31% (95%CI: 1.62–6.61), 1.06% (95%CI: 0.61–1.84) and 2.56% (95%CI: 0.90–7.05). The prevalence of anti-thyroperoxidase antibody was estimated to be 11.68% (95%CI: 7.92–16.89).

**Conclusion:**

The results of this meta-analysis showed a high prevalence of thyroid disorders, especially hypothyroidism. The decision to recommend thyroid screening during pregnancy for all women is still under debate, because the positive effects of treatment on pregnancy outcomes must be ensured. On the other hand, evidence about the effect of thyroid screening and treatment of thyroid disorders on pregnancy outcomes is still insufficient. Nevertheless, a large percentage of general practitioners, obstetricians and gynecologists perform screening procedures in Iran.

## Background

Hypothyroidism during pregnancy has negative effects on the mother and baby. Treatment or non-treatment of mothers have had a profound impact on their children’s future intellectual development [1]. Hypothyroidism during pregnancy is associated with adverse outcomes for both mother and fetus. Specifically, pregnant women with hypothyroidism and even subclinical types are at increased risk for experiencing complications such as recurrent pregnancy loss, neonatal death, preeclampsia, placental abruption, gestational hypertension, gestational diabetes, low birth weight (LBW), preterm birth, fetal distress, intrauterine fetal demise, and deteriorated intellectual function [2–8].

Pregnancy has profound physiological effects on thyroid gland function [9]. During pregnancy, the thyroid gland size increases by 10% in countries with adequate iodine and to a greater extent in countries with iodine deficiency [10]. Thyroid hormone deficiency and iodine requirement both increase approximately by 50% as part of physiology during pregnancy [11]. In addition, pregnancy is a stressful condition for the thyroid gland, resulting in hypothyroidism in women with limited thyroid reserve or iodine deficiency.

The incidence of hyperthyroidism is much lower than hypothyroidism. In 2 to 5 cases per 1000 pregnancies, non-treatment is significantly associated with a higher frequency of labor complications such as preeclampsia, preterm birth, LBW, fetal death and perinatal loss [12].

Nevertheless, routine screening for hypothyroidism during pregnancy is a controversial subject. However, some advocates have recommended it [13]. The American College of Obstetricians and Gynecologists (ACOG) not recommends routine screening for thyroid function in during pregnancy, which is due to insufficient evidence regarding the effect of hypothyroidism screening and treatment on pregnancy outcomes [14]. Quite the contrary, the American Thyroid Association (ATA) has recommended targeted high-risk case finding screening, and laboratory screening limits thyroid-stimulating hormone (TSH) levels only to high-risk cases [1].

A recent report by Nazarpour et al. [15] showed that according to the screening checklist for high-risk cases in thyroid screening during pregnancy, which was suggested by the ATA, over 35% of pregnant women with thyroid dysfunction are overlooked.

Most sources available for assessing the prevalence and outcomes of hypothyroidism during pregnancy are based on populations in Western countries. However, thyroid function is associated with iodine intake, environmental factors, diet, habitat, and genetic susceptibility that may vary between populations. It is therefore necessary to estimate the prevalence of thyroid dysfunction in different populations [16].

Several studies have been performed in regard with thyroid dysfunction in pregnant women in Iran [15, 17–34]. Nevertheless, these studies revealed significantly different results, with the prevalence of hypothyroidism ranging from 0.4 to 34.4% and hyperthyroidism from 0.7 to 16.7%. Since meta-analyses combine multiple studies with the same purpose, and they can provide a more reliable estimate by increasing the sample size and reducing the confidence interval [35–37], the present meta-analysis was conducted to evaluate thyroid dysfunction in Iranian pregnant women.

## Method

### Study protocol

The research steps, including literature search strategy, study selection, data extraction and outcome report were performed according to the Meta-analyses Of Observational Studies in Epidemiology (MOOSE) protocol [37], and finally, reports were provided based on the Preferred Reporting Items for Systematic Review and Meta-analysis (PRISMA) guidelines (Additional file 1) [38]. Each step of the study was performed by two independent authors. Disagreements were resolved through discussion or the involvement of a third author (JH). We registered this review at PROSPERO (registration number: CRD42020166655), available at: https://www.crd.york.ac.uk/PROSPERO/display_record.php?RecordID=166655.

### Search strategy

The PROSPERO database and the national and international databases were first reviewed to find relevant published or ongoing projects. After ensuring the absence of a previous similar study until October 1, 2019, the international online databases, including Web of Science, Ovid, Science Direct, Scopus, EMBASE, PubMed/Medline, Cochrane Library (Cochrane Database of Systematic Reviews - CDSR), EBSCO, CINAHL, as well as national databases, including Magiran (http://www.magiran.com/), Scientific Information Database (SID) (http://www.sid.ir/), Regional Information Center for Science and Technology (RICST) (http://en.ricest.ac.ir/), Barakat Knowledge Network System (http://health.barakatkns.com), Civilica (https://www.civilica.com/), Iranian National Library (http://www.nlai.ir/) Iranian Research Institute for Information Science and Technology (IranDoc ((https://irandoc.ac.ir), and elmnet (https://elmnet.ir) were reviewed. Google Scholar was also used to retrieve online articles on the subjects that might have been missed from online databases to increase the comprehensiveness of the search. References of all remained articles were manually assessed to identify all potential studies. Conference abstracts were also reviewed in case of having useful information. Combined search was done based on Medical Subject Headings (MeSH) keywords such as “prevalence”, “epidemiology”, “frequency”, “incidence”, “pregnant”, “gestational”, “pregnancy”, “prenatal care”, “thyroid”, “hypothyroidism”, “hyperthyroidism”, “peroxidase antibody”, “TPOAb”, and “Iran” using Boolean operators (AND/ OR). Finally, the references in the retrieved articles were also reviewed. An example of combined search in PubMed database is as follows: (prevalence OR epidemiology OR frequency OR incidence) [All terms] AND (pregnant OR gestational OR pregnancy OR prenatal care) [All terms] AND (thyroid OR hypothyroidism OR hyperthyroidism OR peroxidase antibody OR TPOAb) [All terms] AND (Iran) [Affiliation].

### Inclusion criteria

Inclusion criteria were all epidemiologic studies aimed at assessing thyroid dysfunction in pregnant women in English and Persian languages without limitation in publication date according to PICO (Patient, Population, or Problem; Intervention, Prognostic Factor, or Exposure; Comparison or Intervention [if appropriate]; Outcome) [39]: (1) **P**opulation: all Iranian pregnant women population, in all age ranges; (2) **I**ntervention: diagnosis of any thyroid dysfunction by laboratory results for confirmed thyroid dysfunction; (3) **C**omparison: variable aimed for prevalence of thyroid dysfunction, hypothyroidism, and hyperthyroidism such as geographical area, sample size, year of the study, quality of studies and etc.; (4) **O**utcome: Estimate the prevalence of overall thyroid dysfunction, hypothyroidism, and hyperthyroidism.

### Exclusion criteria

Exclusion criteria were as follows: (1) non-random sampling, (2) duplicate studies, (3) non-Iranian studies, (4) population other than pregnant women, (5) not relevant to the target subject, (6) participants with specific diseases (e.g., gestational diabetes), (7) unspecified diagnostic intervention, (8) poor qualitative evaluation, and (9) case reports, review articles, letters to the editor without qualitative data.

### Study selection and data extraction

Two reviewers (M.A and M.Sh) independently reviewed the titles and abstracts of all identified records, and at this point, duplicate and unrelated studies were excluded. Duplicate articles were identified manually or using EndNote X7 Application. Then, both reviewers screened the articles independently to review eligible studies according to inclusion and exclusion criteria. Both reviewers independently extracted the data from the articles. Any disagreement between the data extractors was resolved by consensus or by the third author.

Data summary form was prepared in Microsoft Excel sheet, which included: First author’s name, year of publication, year of the study, study design, region, mean age and standard deviation, mean gestational age and standard deviation, trimester of pregnancy, method of diagnosing thyroid dysfunction and cut-off point for each of the tests, sampling technique, sample size, number of positive thyroid dysfunction cases, number of positive cases of hypothyroidism (clinical and subclinical) and hyperthyroidism (clinical and subthreshold), and number of positive cases of anti-thyroid peroxidase antibody.

### Definitions

In this study, thyroid disorder was defined as all cases of positive hypothyroidism and hyperthyroidism.

### Qualitative evaluation

The adapted Newcastle-Ottawa Scale (NOS) was used for cross-sectional studies to evaluate the quality of studies [40]. The maximum attainable score was 9. Three categories, including scores below 6, scores 6 to 7, and scores 8 to 9 were respectively defined as low, medium and high quality for the studies.

### Statistical analysis

I^2^ index and Q test were used to assess differences in studies. Its value may vary from 0 to 100% and the values of 75, 50 and 25% respectively indicate high, medium and low heterogeneity among studies [41, 42]. In addition, *P* < 0.1 was used to determine heterogeneity. Considering the high heterogeneity of the studies, we performed a meta-analysis based on the random effects model and reported the results based on pooled prevalence and 95% confidence interval (CI). Diagnosis of heterogeneity between studies was performed based on meta-regression and subgroup analysis. Sensitivity analysis was performed by omitting one study at a time to evaluate the consistency of the results. Funnel plots and Begg and Egger’s tests were used to evaluate publication bias [43, 44]. All analyses were performed using Comprehensive Meta-Analysis Software ver 2. *P*-value less than 0.05 was considered statistically significant.

## Results

### Description of included studies

**Fig. 1:**
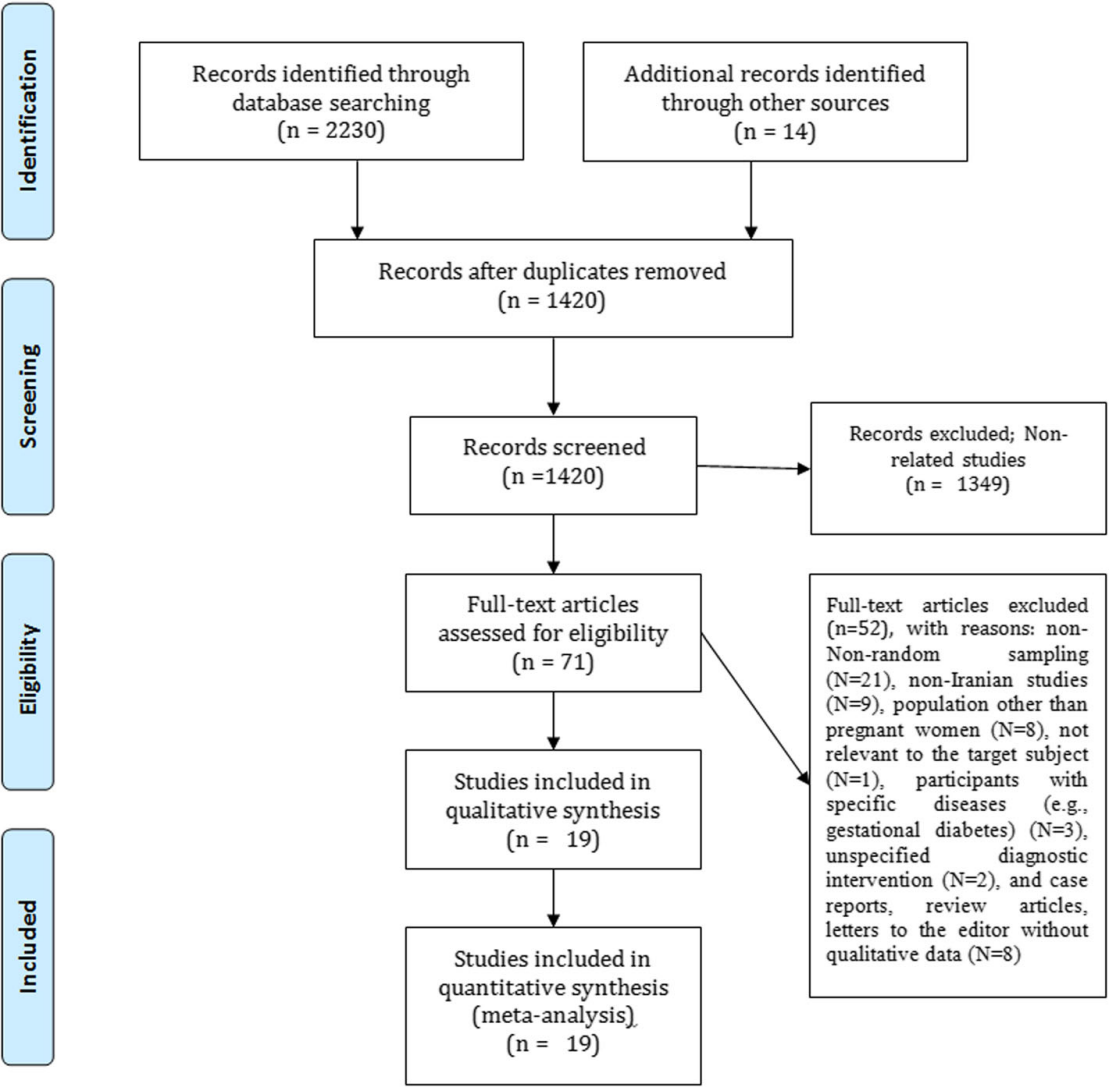
PRISMA flowchart

We identified 2244 potential articles from the databases, and 1420 articles remained after removing the duplicate and unrelated studies. After evaluating the full text, 52 articles were removed for at least one of the following reasons: non-random sampling (*n* = 21), non-Iranian studies (*n* = 9), study population other than pregnant women (*n* = 8), sample size smaller than 100 participants (*n* = 1), participants with specific diseases (e.g., gestational diabetes) (*n* = 3), unspecified diagnostic intervention (*n* = 2), poor qualitative evaluation (*n* = 0), and case reports, review articles, letters to the editor without qualitative data (*n* = 8). This process is illustrated in Fig. 1. Finally, 19 eligible studies were used for meta-analysis (Table 1). The mean age of the study participants was 26.73 (95% CI: 25.89–27.56) years.

**Table 1:** Summary of characteristics in studies into a meta-analysis

Ref.	First author, Published Year	Year	Place	Number	GA^**a**^	Method	Criteria	Quality score
[17]	Naghshineh E, 2012	2010–11	Isfahan	1057	NR	NR	Hypothyroidism: TSH > 2.50 mIU/L	7
[18]	Mansourian AR, 2010	2007–08	Gorgan	120	First trimester	ELISA method for TSH	Hyperthyroidism: TSH < 0.32 mIU/L	7
[19]	Naderi T, 2012	2010	Kerman	620	< 20 week	ELISA method for TSH and fT4	Clinical hypothyroidism: TSH > 4 mIU/L; subclinical hypothyroidism: 2 < TSH < 4 mIU/L and FT4 < 0.07 ng/dL	8
[20]	Dehghani Zahedani M, 2010	2007–08	BandarAbbas	608	All trimester	RIA method for TSH and ELISA method for Anti-TPO Ab	clinical hypothyroidism: TSH > 3.5 mIU/L and FT4 < 1.3 ng/dL; subclinical hypothyroidism: TSH > 3.5 mIU/L and FT4 > 1.3 ng/dL; hyperthyroidism: TSH < 0.3 mIU/L and FT4 > 4.6 ng/dL; TPOAb positive: > 75 IU/mL	8
[21]	Borzouei Sh, 2019	2015–16	Hamadan	852	First trimester	RIA method for T4, IRMA for TSH and ELISA for anti-Anti-TPO Ab	Clinical hypothyroidism: TSH > 2.5 mIU/L and low FT4 or TSH ≥ 10 mIU/L; subclinical hypothyroidism: TSH > 2.5 mIU/L and normal FT4; subclinical hyperthyroidism: TSH < 0.1 mIU/L and normal (FT4 and FT3); subclinical hyperthyroidism: TSH < 0.1 mIU/L and high (FT4 or FT3); Anti-TPO Ab positive: > 40 IU/mL	7
[22]	Rahmatelahi M, 2016	2016–17	Shahrood	369	< 20 week	NR	Clinical hypothyroidism: TSH > 4 mIU/L with low FT4; Subclinical hypothyroidism: TSH > 4 mIU/L with normal FT4; clinical hyperthyroidism: TSH < 0.4 mIU/L and elevated FT4; subclinical hyperthyroidism: TSH level < 0.4 mIU/L and with normal FT4	7
[23]	Saki F, 2014	2011–12	Shiraz	586	15–18 week	ECL method for TSH and T4	Clinical hypothyroidism: TSH > 3 mIU/L and low FT4 or TSH ≥ 10 mIU/L; 3 < TSH < 10 mIU/L and normal FT4; clinical hyperthyroidism: TSH < 0.2 mIU/L and elevated FT4 or TSH < 0.1; subclinical hyperthyroidism: 0.1 ≥ TSH ≥ 0.2 mIU/L and normal FT4	8
[24]	Lotfalizadeh M, 2017	2012–13	Mashhad	1000	First trimester	RIA method for TSH and ELISA method for FT4	Hypothyroidism: TSH > 3 mIU/L	7
[25]	Yassaee F, 2014	2008–12	Tehran	3158	NR	CLIA method for TSH and T4	Clinical hypothyroidism: TSH > 2.5 mIU/L in the first trimester or TSH > 3 mIU/L in the second or third trimester, with normal FT4 (0.8–1.7 ng/dL); TSH > 2.5 mIU/L and FT4 < 0.8 ng/dL	8
[26]	Mehran L, 2013	2004–06	Tehran	299	All trimester	RIA method for of TT4 and TT3 and IRMA method for TSH	Anti-TPO Ab: > 40 IU/mL	8
[27]	Moradi S, 2013	2012	Tehran	584	All trimester	IRMA for TSH and by RIA for FT4, T4, T3,T3RU and anti-TPO	The reference range for TSH is 0.2–2.5 mIU/L in the first trimester and 0.3–3.0 mIU/L in the third trimester.	9
[15]	Nazarpour S, 2016	2013–14	Tehran	1480	NR	RIA and IRMA methods T4 for TSH	Clinical hypothyroidism: 5 < TSH < 10 mIU/L and FT4 <1 ng/dL or TSH ≥ 10 mIU/L; subclinical hypothyroidism: 2.5 < TSH < 10 mIU/L and 1 < FT4 < 4.5 ng/dL; clinical hyperthyroidism: TSH < 0.02 mIU/L and FT4 > 4.5 ng/dL; subclinical hyperthyroidism: TSH < 0.02 mIU/L and 1< FT4 < 4.5 ng/dL; Anti-TPO Ab positive: > 50 mIU/L	8
[28]	Kianpour M, 2019	2017	Isfahan	418	First trimester	NR	hypothyroidism: TSH > 2.5 mIU/L; hyperthyroidism: TSH < 0.1 mIU/L; Anti-TPO Ab positive: > 60 mIU/L	8
[29]	Taghavi M, 2009	2006–08	Mashhad	500	First trimester	RIA method for TSH, FT4 and FT3	TSH level > 4 mIU/L and reduced FT4 concentration as clinical hypothyroidism; TSH level > 4 mIU/L and normal serum FT4 concentration as clinical hypothyroidism; TSH level < 0.4 mIU/L and elevated FT4 concentration as clinical hyperthyroidism	8
[30]	Nazarpour S, 2018	2013–16	Tehran	1843	First trimester	RIA method for T4 and IRMA method for TSH and IEMA method for Anti-TPO Ab	Clinical hyperthyroidism: TSH < 0.1 mIU/L and FT4 > 4.5 ng/dL; clinical hypothyroidism: TSH > 10 mIU/L or TSH > 2.5 mIU/L and FT4I < 1 ng/dL; subclinical hypothyroidism: elevated TSH (2.5–10 mIU/L) and normal FT4I (1–4.5 ng/dL); Anti-TPO Ab-positive: > 50 mIU/L	9
[31]	Zangeneh M, 2015	2011–12	Kermanshah	1200	< 16 week	ELISA method	Reference renege for TSH is 0.27–4.2 mIU/L and for FT4I is 5.13–14.6	7
[32]	Maleki N, 2014	2011–3	Bushehr	313	24–28 week	RIA method for FT4 and FT3	Reference renege in the first, second and third trimesters for TSH 0.1–2.5 mIU/L, 0.2–3.0 mIU/L; and 0.3–3.0 mIU/L, respectively.	7
[33]	Sarkhail P, 2016	2004–06	Tehran	120	All trimester	RIA method for TT4 and TT3 and IRMA for TSH	Reference renege in the first, second and third trimesters are TSH (0.2–3.9, 0.5–4.1, and 0.6–4.1 mIU/l); TT4 (8.2–18.5, 10.1–20.6, and 9–19.4 ≥ g/dL); and TT3 (137–278, 154–327, and 137–323 ng/dL), respectively.	7
[34]	Mellati Ali Avesti SF, 2003	2002	Zanjan	500	First trimester	IRMA method for TSH and RIA method for FT4	NR	6

*NR Not reported, TSH thyroid-stimulating hormone, mIU/L Milli-international units per litre, T3 triiodothyronine, T4  Thyroxine,GA* Gestational age, *ELISA* Enzyme-linked immunosorbent assay, *RIA* Radioimmunoassay, *IRMA* Immunoradiometric assay, *CLIA* Chemiluminescent immunoassay, *IEMA* immunoenzymometric assay, *anti-TPO Ab anti-thyroperoxidase antibody*

### Thyroid dysfunction

**Fig. 2:**
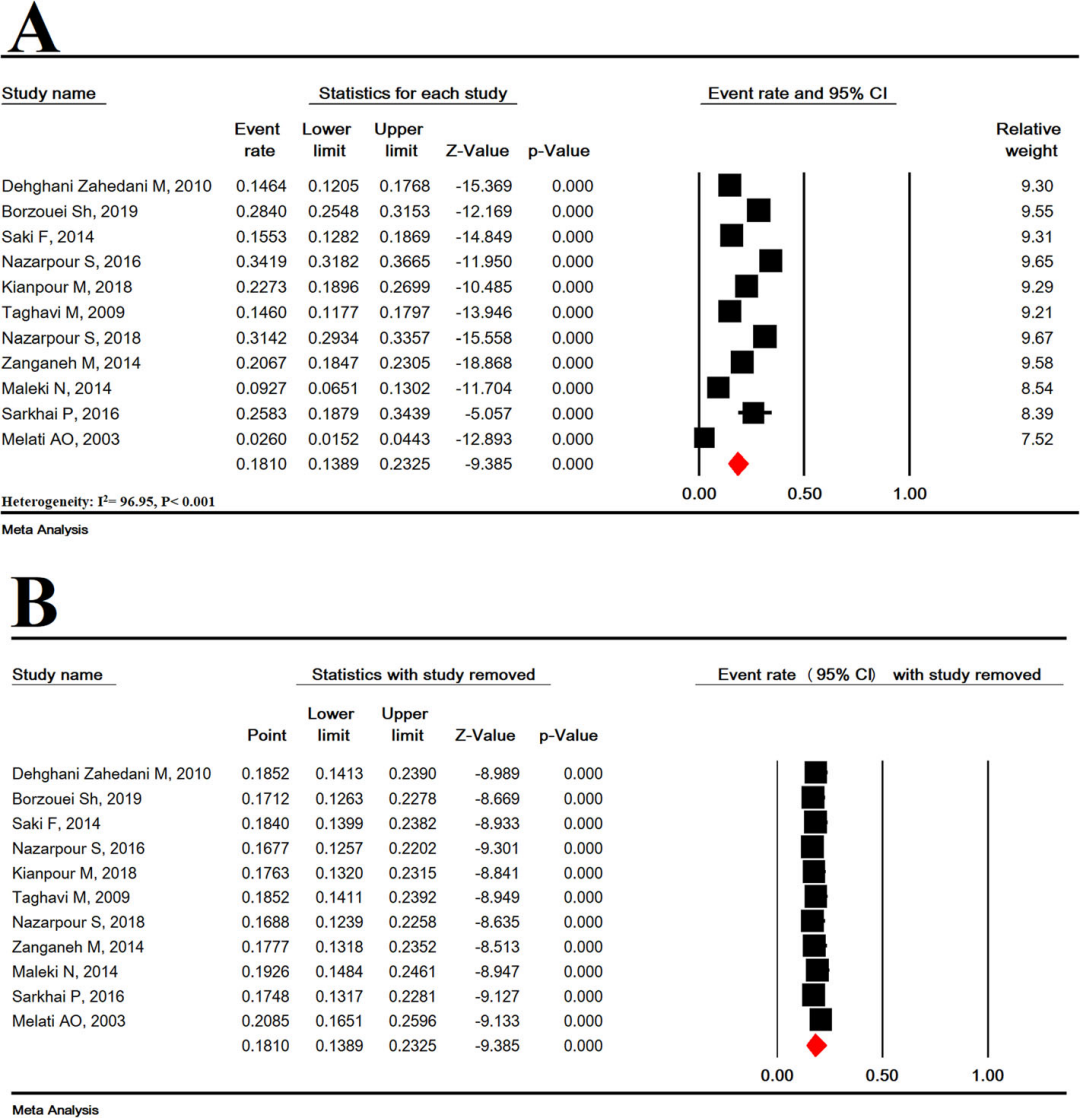
Prevalence of thyroid dysfunction in pregnant Iranian women (**a**) and sensitivity analysis (**b**)

The prevalence of thyroid dysfunction in 8420 Iranian pregnant women was 18.10% (95% CI: 13.89–23.25) in 11 studies. Heterogeneity was high among the studies: (Heterogeneity: I^2^ = 96.95%, *P* < 0.001) (Fig. 2a). Sensitivity analysis by omitting one study showed that the overall estimate is robust (Fig. 2b).

### Subgroup analysis of thyroid dysfunction

Subgroup analysis of thyroid dysfunction was significant in terms of geographical area (*P* = 0.014), year of the study (*P* < 0.001) and sample size (*P* = 0.020), but was not significant in terms of quality of studies (*P* = 0.177) (Additional file 2).

### Hypothyroidism

**Fig. 3:**
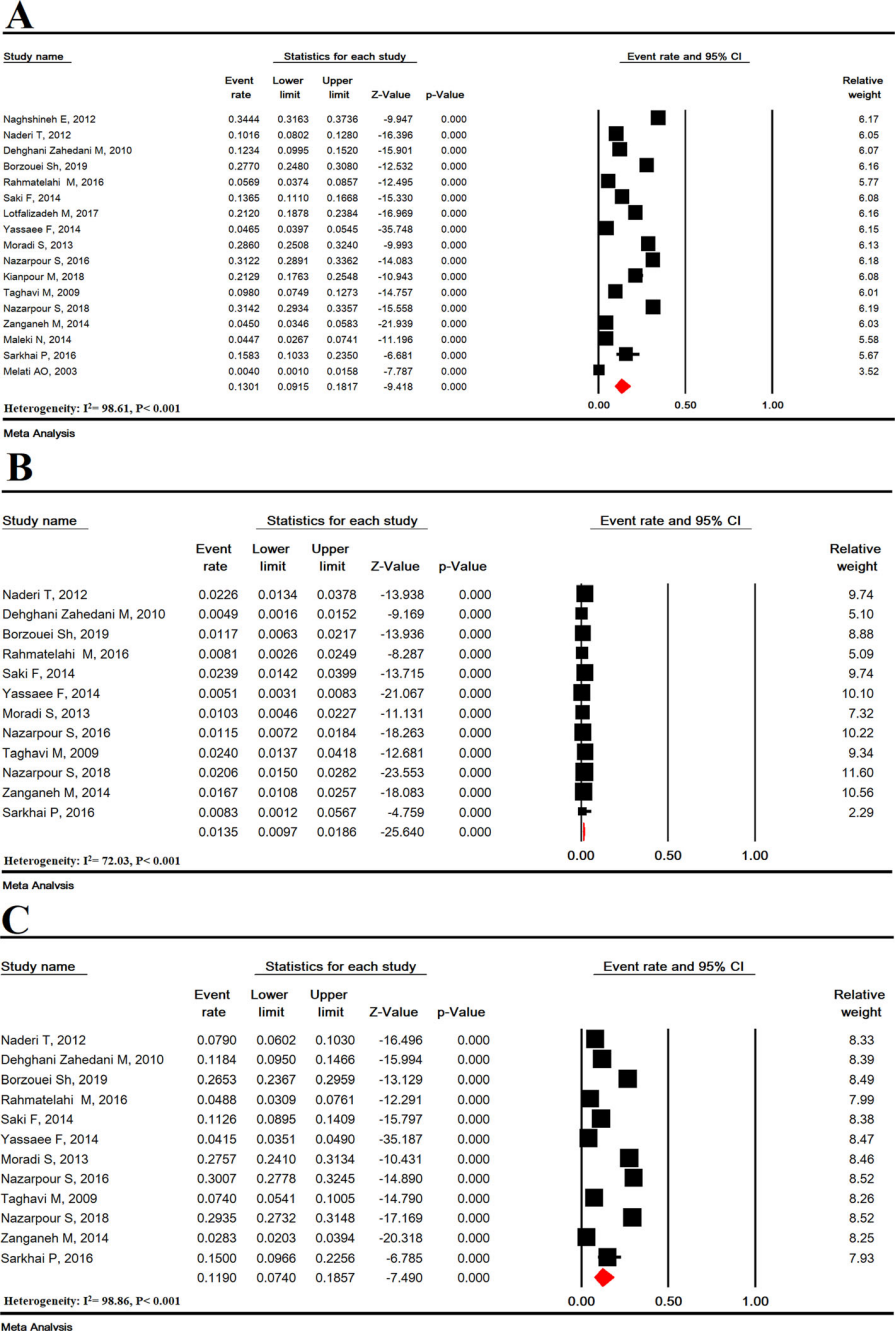
Prevalence of hypothyroidism (**a**), clinical hypothyroidism (**b**), subclinical hypothyroidism in pregnant Iranian women

The prevalence of hypothyroidism (in 17 studies with a sample size of 15,208 people), clinical hypothyroidism (in 12 studies with a sample size of 11,920 people), and subclinical hypothyroidism (in 12 studies with a sample size of 11,920 people) in Iranian pregnant women was respectively estimated to be 13.01% (95% CI: 9.15–18.17), 1.35% (95% CI: 0.97–1.86) and 11.90% (95% CI: 7.40–18.57) (Fig. 3). Sensitivity analysis by omitting one study showed the overall results to be robust (Additional file 3).

### Subgroup analysis of hypothyroidism

Subgroup analysis of hypothyroidism prevalence was significant in terms of year of the study (*P* = 0.003) and sample size (*P* = 0.043), but was not significant in terms of geographical area (*P* = 0.573) and quality of studies (*P* = 0.210) (Additional file 4).

Subgroup analysis of clinical hypothyroidism prevalence was not significant in terms of geographic regions (*P* = 0.210), year of the study (*P* = 0.944), sample size (*P* = 0.885) and quality of studies (*P* = 0.132) (Additional file 5).

Subgroup analysis of subclinical hypothyroidism prevalence was significant in terms of year of the study (*P* = 0.006) and but was not significant in terms of geographical area (*P* = 0.196), sample size (*P* = 0.249) and quality of studies (*P* = 0.560) (Additional file 6).

### Hyperthyroidism

**Fig. 4:**
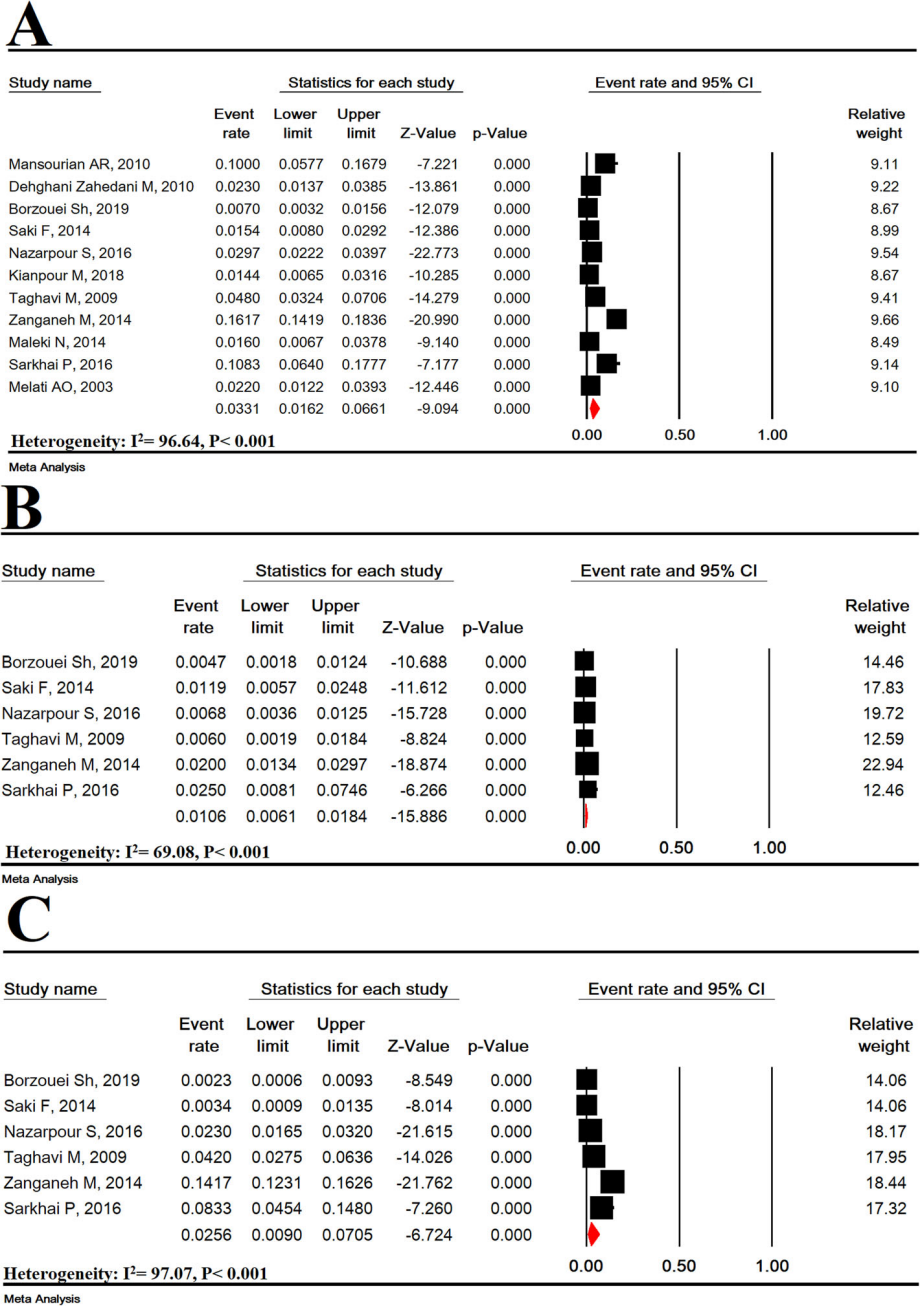
Prevalence of hyperthyroidism (**a**), clinical hyperthyroidism (**b**), subclinical hyperthyroidism in pregnant Iranian women

The prevalence of hyperthyroidism (in 11 studies with a sample size of 6697 people), clinical hyperthyroidism (in 6 studies with a sample size of 4738 people), and subclinical hyperthyroidism (in 6 studies with a sample size of 4738 people) in Iranian pregnant women was respectively estimated to be 3.31% (95% CI: 1.62–6.61), 1.06% (95% CI: 0.61–1.84) and 2.56% (95% CI: 0.90–7.05) (Fig. 4). Sensitivity analysis by omitting one study demonstrated the overall results to be robust (Additional file 7).

### Subgroup analysis of hyperthyroidism

Subgroup analysis of hyperthyroidism prevalence was statistically significant in terms of geographical area (*P* < 0.001) and sample size (*P* = 0.048), but was not significant in terms of year of the study (*P* = 0.118) and quality of studies (*P* = 0.346) (Additional file 8).

### The prevalence of thyroid peroxidase

**Fig. 5:**
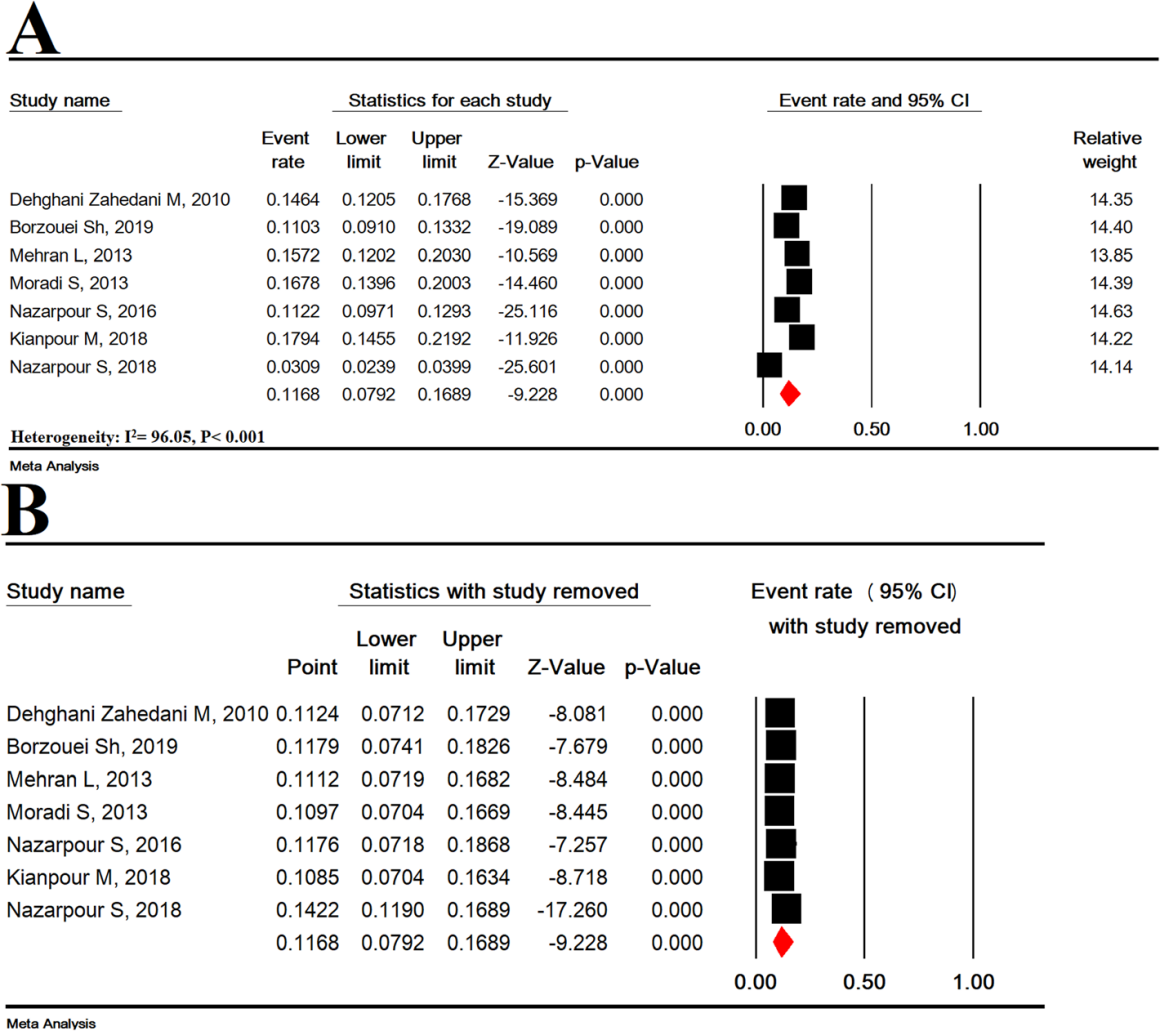
Prevalence of anti-thyroperoxidase antibody (**a**) and sensitivity analysis (**b**) in pregnant Iranian women

The prevalence of anti-thyroperoxidase antibody (anti-TPO Ab) in 6084 Iranian pregnant women was estimated to be 11.68% (95% CI: 7.92–16.89) (Fig. 5).

### Meta-regression

**Fig. 6:**
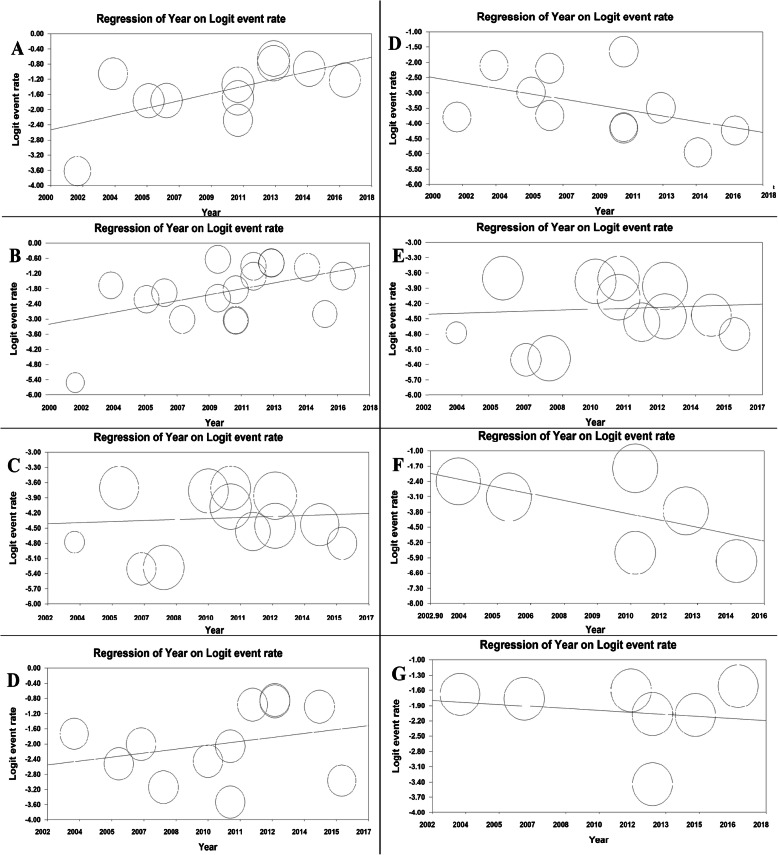
Meta-regression for prevalence of thyroid function disorder (**a**), hypothyroidism (**b**), clinical hypothyroidism (**c**), subclinical hypothyroidism (**d**), hyperthyroidism (**e**), clinical hyperthyroidism (**f**), subclinical hyperthyroidism (**g**) and anti-thyroperoxidase antibody (h) in pregnant Iranian women

Meta-regression model in terms of year of the study was significant for the prevalence of thyroid dysfunction (meta-regression coefficient: 0.106, 95% CI 0.049 to 0.164, *P* < 0.001) and hypothyroidism (meta-regression coefficient: 0.129, 95% CI 0.034 to 0.225, *P* = 0.007) but was not significant for clinical hypothyroidism (meta-regression coefficient: 0.014, 95% CI -0.099 to 0.128, *P* = 0.806), subclinical hypothyroidism (meta-regression coefficient: 0.071, 95% CI -0.043 to 0.187, *P* = 0.221), hyperthyroidism (meta-regression coefficient: -0.100, 95% CI -0.275 to 0.073, *P* = 0.257), clinical hyperthyroidism (meta-regression coefficient: -0.087, 95% CI -0.245 to 0.070, *P* = 0.276), subclinical hyperthyroidism (meta-regression coefficient: -0.234, 95% CI -0.542 to 0.072, P = 0.134), and anti-TPO Ab (meta-regression coefficient: -0.0255, 95% CI -0.134to 0.083, P = 0.646) (Fig. 6).

### Publication bias

The publication bias for the studies is shown in Additional file 9. Significance levels were also calculated for thyroid dysfunction (Begg = 0.029 and Egger’s = 0.001), hypothyroidism (Begg = 0.003 and Egger’s = 0.004), clinical hypothyroidism (Begg = 0.029 and Egger’s = 0.001), subclinical hypothyroidism (Begg = 0.058 and Egger’s = 0.010), hyperthyroidism (Begg = 0.533 and Egger’s = 0.002), clinical hyperthyroidism (Begg = 0.999 and Egger’s = 0.269), subclinical hyperthyroidism (Begg = 0.763 and Egger’s = 0.026), and anti-TPO Ab (Begg = 0.710 and Egger’s = 0.368).

## Discussion

This study is the first systematic review and meta-analysis about thyroid disorders in Iranian pregnant women to date. The prevalence of thyroid dysfunction in Iranian pregnant women was estimated to be 18.10%. The prevalence of thyroid dysfunction was reported to be varied in other studies in China (10.2–15.6%) [45, 46], Spain (16.6%) [47], India (13.25%) [48], and Belgium (15.3%) [49]. Thyroid dysfunction is the second endocrine disorder (after diabetes mellitus) that affects women of reproductive age [50]. Thyroid disorders are associated with a number of diseases such as celiac disease, dermatitis, chronic hepatitis, lupus, Addison’s disease, ovarian insufficiency, rheumatoid arthritis, type 1 diabetes, while various factors such as smoking, birth control pills, some medications, history of ovarian cyst, diet, autoimmune factors, radiation, and thrombocytopenia are among the predisposing factors [51–53].

The reported data on the function of thyroid during pregnancy varies according to epidemiological, environmental and analytical factors. Recent studies have shown that thyroid disorders may occur more frequently than previously estimated [45, 47, 49, 54–58]. In the present study, the prevalence of hypothyroidism was 13.01% (overt [1.35%] and subclinical [11.90%]). The prevalence of hypothyroidism during pregnancy ranges from 2.5–3% [56, 57] to 6.3–7.5% [45, 54] in different studies, yet it reached 15.5% in a recent study by Blatt in United States [55]. The prevalence of overt hypothyroidism has been reported in 0.3–1.9% of pregnancies and subclinical hypothyroidism has been reported in approximately 5.5% of pregnancies [58, 59].

It is important to note the cutoff points defined for hypothyroidism; according to one study, if cutoff value of above 4.5 mIU was used for TSH test, the incidence of hypothyroidism was 5.5%, whereas using cutoff levels above 2.5 mIU [60] showed that 27.6% of women were positive in terms of hypothyroidism, which is five times higher. This highlights the importance of using specific biochemical parameters to properly evaluate thyroid function in pregnancy [47]. In our meta-analysis, pooled estimate of thyroid dysfunction is conditioned by the utilization of specific cut-off values, according to the current guidelines endorse the use of specific reference ranges.

In the present study, the prevalence of hyperthyroidism was estimated to be 3.31% (overt [1.06%] and subclinical [2.56%]) in the present study. Other studies have reported that hyperthyroidism occurs in about 0.2–1.0% of all pregnancies, with overt hyperthyroidism occurring in 0.1 to 0.4% of pregnancies and subclinical in up to 1% of pregnancies [12, 48, 61–63].

Undesirable effects of thyroid disorders on the outcomes of pregnancy have been shown in various studies [3, 53, 64]. However, the effectiveness of treatment has also become a challenge. Recent meta-analyses investigating the therapeutic effect of levothyroxine supplementation on pregnancy outcomes have shown that miscarriage rates and preterm birth rates are reduced in women with subclinical hypothyroidism or autoimmune thyroid diseases [64, 65]. Another systematic review and meta-analysis showed that levothyroxine treatment significantly improved pregnancy outcomes (such as delivery rates) and significantly reduced pregnancy complications (such as miscarriage) [66] in women with subclinical hypothyroidism undergoing assisted reproductive technology. The results of the study by Abalovich [52] et al. show that the prenatal development does not depend on whether the hypothyroidism is overt or subclinical but depends on the type of treatment received. Effective treatment of hypothyroidism during pregnancy minimizes the risks and, in general, makes it possible to have pregnancy without complications. On the other hand, some other systematic reviews where levothyroxine has not been effective such as Akhtar et al. [67].

Public screening for thyroid disease before or during pregnancy is still controversial. For screening to be recommended, the disease must be prevalent, it must show adverse health consequences and must be curable. According to the guidelines of the ATA, there is insufficient evidence to recommend public screening for abnormal TSH concentrations in early stages of pregnancy. Therefore, this association recommends targeted TSH test if any of the following risk factors are identified in all patients who intend to become pregnant or have recently become pregnant: 1. Hypothyroidism/hyperthyroidism or symptoms/signs of thyroid dysfunction; 2. Positive type of thyroid antibody or detection of goiter; 3. History of radiation therapy to the head and neck or previous thyroid surgery; 4. Over 30 years of age; 5. Type 1 diabetes or other autoimmune disorders; 6. History of miscarriage, preterm labor or infertility; 7. Multiple previous pregnancies (≥2); 8. Family history of autoimmune thyroid disease or thyroid dysfunction; 9. Morbid obesity (Body Mass Index (BMI) ≥ 40 kg/m^2^); 10. Use of amiodarone or lithium, or recent administration of radiographic iodinated contrast; and 11. Residence in a region known for moderate to severe iodine deficiency [68]. Compared to global screening, the case-finding strategy has a low sensitivity, in which 30 to 80% of women with hypothyroidism are missed, and this has been confirmed in numerous studies and many women suffering from isolated hypothyroxinemia are not identified [54, 56, 69].

According to the guidelines of European Thyroid Association in 2014, most authors recommend global screening for the beneficial therapeutic effects of hypothyroidism, and emphasize on the fact that a targeted approach leads to the detection of a high percentage of women with subclinical hypothyroidism [70]. The Spanish Society of Endocrinology and Nutrition [13] and the Indian Thyroid Society [71] have advocated global screening in early stages of pregnancy or before pregnancy. A survey of members of professional associations showed that 42.7% of respondents in Latin America and 43% in Europe perform global screening [72, 73], while only 21% of members of the Asia-Oceania Thyroid Association (AOTA) perform this [74], and 74% of ATA members support such an approach [75].

Another important point in public screening is cost-effectiveness; if the usefulness of a screening model is definitively proven, women’s global screening in the first trimester appears to be cost-effective [76–78]. A study by Dosiou et al. [79] demonstrated the cost-effectiveness of universal screening of pregnant women with anti-TPO Ab and TSH antibodies in the first trimester of pregnancy compared to a high-risk screening strategy. Based on sensitivity analyses, even when the benefits of screening were limited to the diagnosis and treatment of overt hypothyroidism, screening was very cost-effective at less than $8000/quality-adjusted life-year [80].

This study has several strengths. It utilized a comprehensive search strategy based on the MOOSE protocol to maximize the possibility of identifying relevant literature. On the other hand, the research was independently carried out by two authors, and the differences were resolved by group discussion. In cases where there was some ambiguity in the article, we contacted the first author or the corresponding author. We used random effects model to evaluate the data to provide a conservative estimate of the prevalence of thyroid disorders, and subgroup analysis and meta-regression model were performed to detect the cause of heterogeneity and to evaluate the publication bias. Finally, the limitation of our study is related to limitations of national databases in combined searches, the lack or absence of studies showing the prevalence of thyroid dysfunction in some geographical areas such as north and east of Iran is another limitation.

There was significant heterogeneity between the studies for thyroid dysfunction and given the available data, we could attribute this difference to geographic area, year of the study and sample size based on subgroup analysis. In addition, the prevalence of hypothyroidism was significantly different according to the year of the study and sample size, whereas the prevalence of subclinical hypothyroidism was dependent on year of the study and hyperthyroidism was dependent on geographical area and sample size, and yet it seems that other differences, such as differences in diagnostic criteria [47] or race [81] are also effective; reviewing these cases was not possible using the data available.

The overall prevalence of thyroid disorders and hypothyroidism during pregnancy in Iran is increasing over time, so it is necessary for Iranian health policymakers to take the necessary intervention measures in this regard.

For future studies, it is recommended that: 1. a cost-effective screening for TSH be performed during pregnancy in the Iranian population. 2. More studies should be conducted to assess the effect of treating thyroid disorders, especially hypothyroidism (clinical and subclinical), on the outcomes of pregnancy for clinical decision-making.

## Conclusion

This study provides policymakers and physicians with comprehensive data regarding the status of thyroid disease. The results of this meta-analysis showed a high prevalence of thyroid disorders, especially hypothyroidism. The decision to recommend thyroid screening during pregnancy for all women is still under debate, because the positive effects of treatment on pregnancy outcomes must be ensured. On the other hand, evidence about the effect of thyroid screening and treatment of thyroid disorders on pregnancy outcomes is still insufficient. Nevertheless, a large percentage of general practitioners, obstetricians and gynecologists perform screening procedures in Iran.

## Supplementary information

**Additional file 1.** PRISMA checklist

**Additional file 2.** Subgroup analysis of thyroid function disorders in pregnant Iranian women based on geographic regions (A), year of studies (B), sample size (C), and quality of studies (D)

**Additional file 3.** Sensitivity analysis for prevalence of hypothyroidism (A), clinical hypothyroidism (B), subclinical hypothyroidism (C) in pregnant Iranian women

**Additional file 4.** Subgroup analysis of hypothyroidism in pregnant Iranian women based on geographic regions (A), year of studies (B), sample size (C), and quality of studies (D)

**Additional file 5.** Subgroup analysis of clinical hypothyroidism in pregnant Iranian women based on geographic regions (A), year of studies (B), sample size (C), and quality of studies (D)

**Additional file 6.** Subgroup analysis of subclinical hypothyroidism in pregnant Iranian women based on geographic regions (A), year of studies (B), sample size (C), and quality of studies (D)

**Additional file 7.** Sensitivity analysis for prevalence of hyperthyroidism (A), clinical hyperthyroidism (B), subclinical hyperthyroidism (C) in pregnant Iranian women

**Additional file 8.** Subgroup analysis of hyperthyroidism in pregnant Iranian women based on geographic regions (A), year of studies (B), sample size (C), and quality of studies (D)

**Additional file 9.** Funnel plot for thyroid function disorder (A), hypothyroidism (B), clinical hypothyroidism (C), subclinical hypothyroidism (D), hyperthyroidism (E), clinical hyperthyroidism (F), subclinical hyperthyroidism (G) and anti TPO (H) in pregnant Iranian women

## Data Availability

Not applicable.

## Abbreviations

ACOG: American College of Obstetricians and Gynecologists
anti-TPO Ab: Anti-Thyroperoxidase Antibody
AOTA: Asia-Oceania Thyroid Association
ATA: American Thyroid Association
BMI: Body Mass Index
CDSR: Cochrane Database of Systematic Reviews
CI: Confidence interval
IranDoc: Iranian Research Institute for Information Science and Technology
LBW: Low birth weight
MeSH: Medical Subject Headings
mIU/L: Milli-international units per litre
MOOSE: Meta-analyses Of Observational Studies in Epidemiology
NOS: Newcastle-Ottawa Scale
NR: Not reported
PICO: Patient, Population, or Problem; Intervention, Prognostic Factor, or Exposure; Comparison or Intervention [if appropriate]; Outcome
PRISMA: Preferred Reporting Items for Systematic Review and Meta-analysis
PROSPERO: International prospective register of systematic reviews
RICST: Regional Information Center for Science and Technology
SID: Scientific Information Database
TSH: Thyroid-stimulating hormone
T3: Triiodothyronine
T4: Thyroxine
