# Supplementary figures and images for: Thyroid dysfunction in Iranian pregnant women: a systematic review and meta-analysis
Source: BMC Pregnancy Childbirth. 2020 Jul 14;20:405. doi: 10.1186/s12884-020-03040-5 (PMC7386166; doi:10.1186/s12884-020-03040-5)

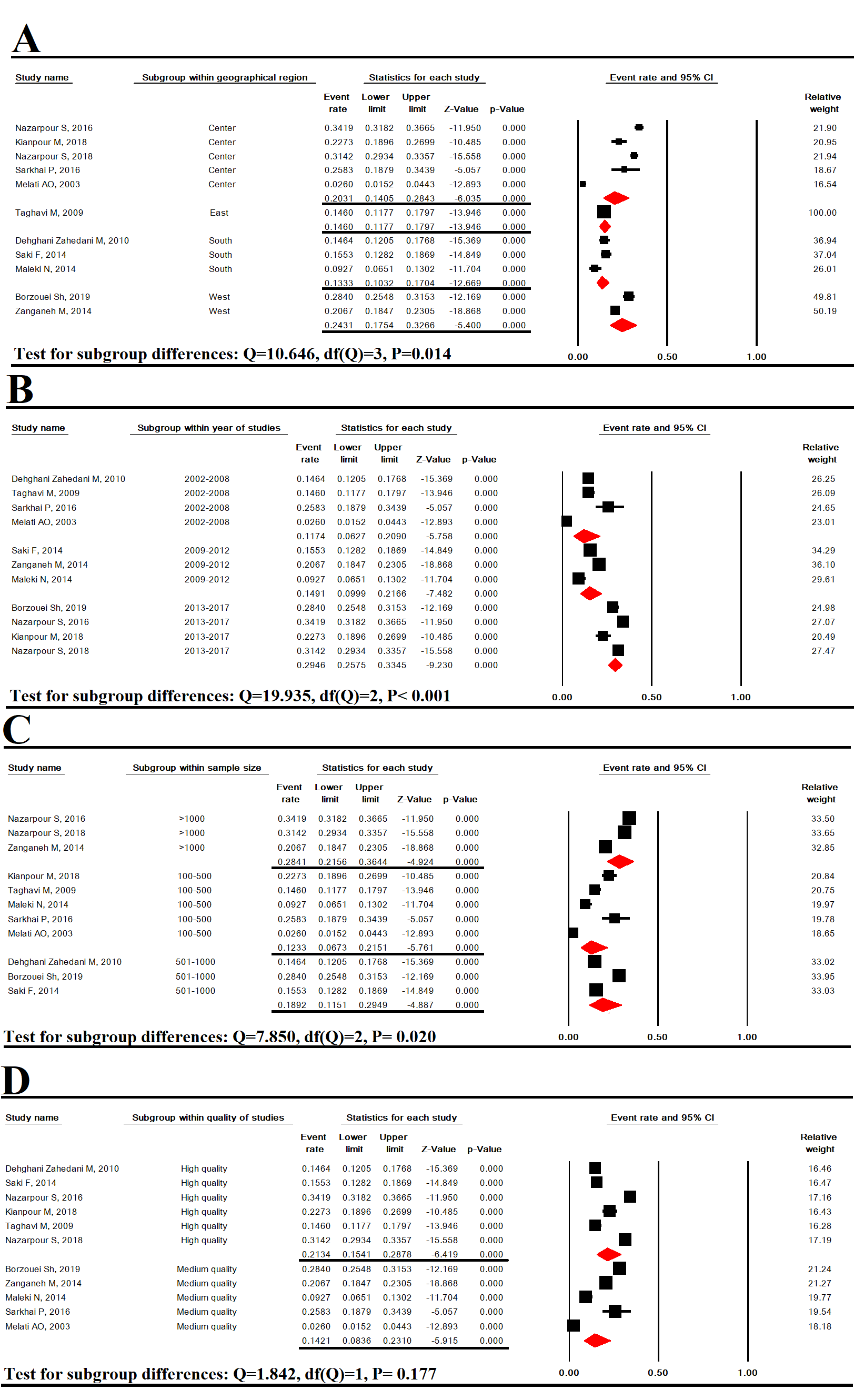

Supplement: Supplementary file 2 — Additional file 2. Subgroup analysis of thyroid function disorders in pregnant Iranian women based on geographic regions (A), year of studies (B), sample size (C), and quality of studies (D) [file 12884_2020_3040_MOESM2_ESM.tif]

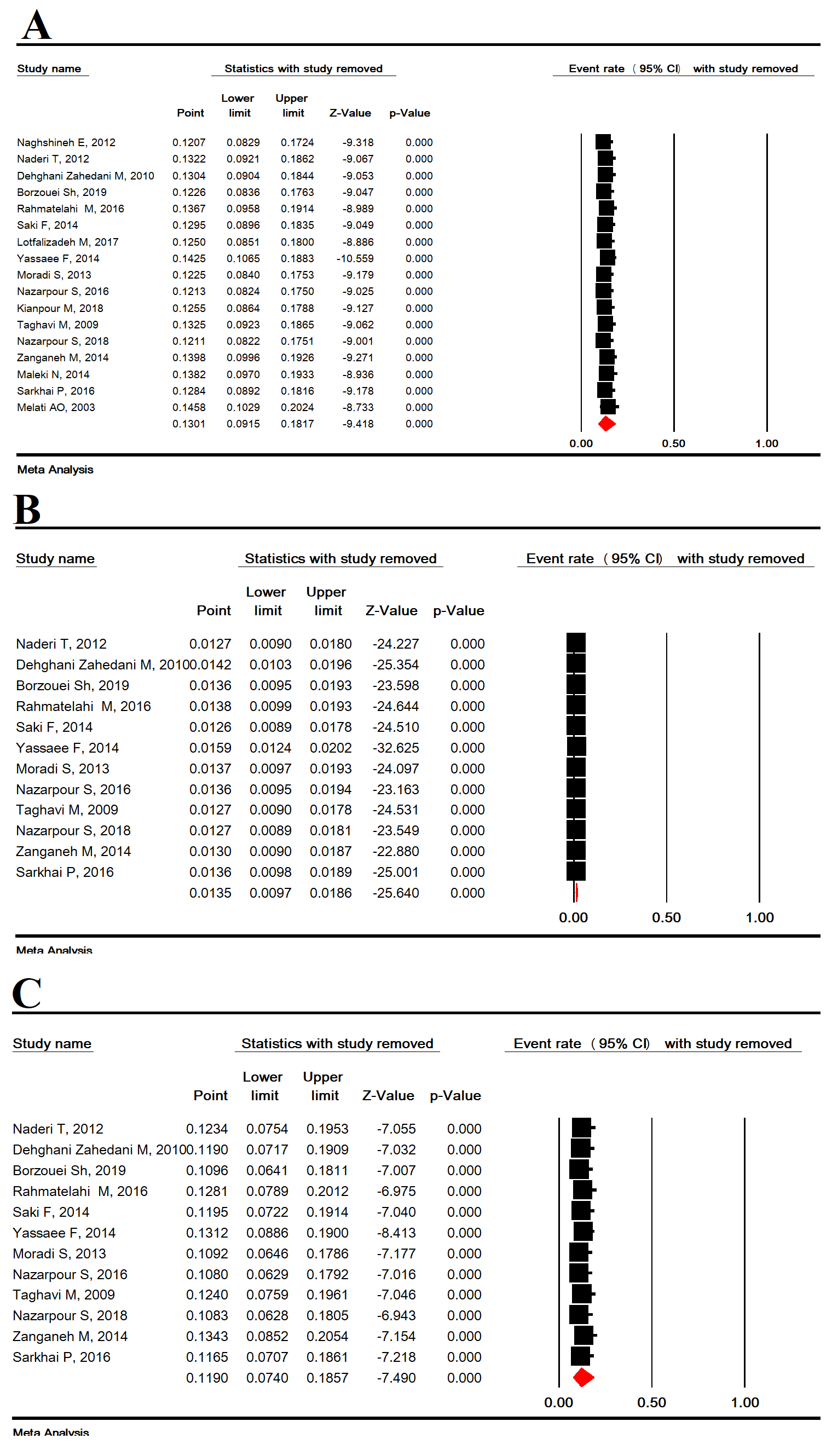

Supplement: Supplementary file 3 — Additional file 3. Sensitivity analysis for prevalence of hypothyroidism (A), clinical hypothyroidism (B), subclinical hypothyroidism (C) in pregnant Iranian women [file 12884_2020_3040_MOESM3_ESM.tif]

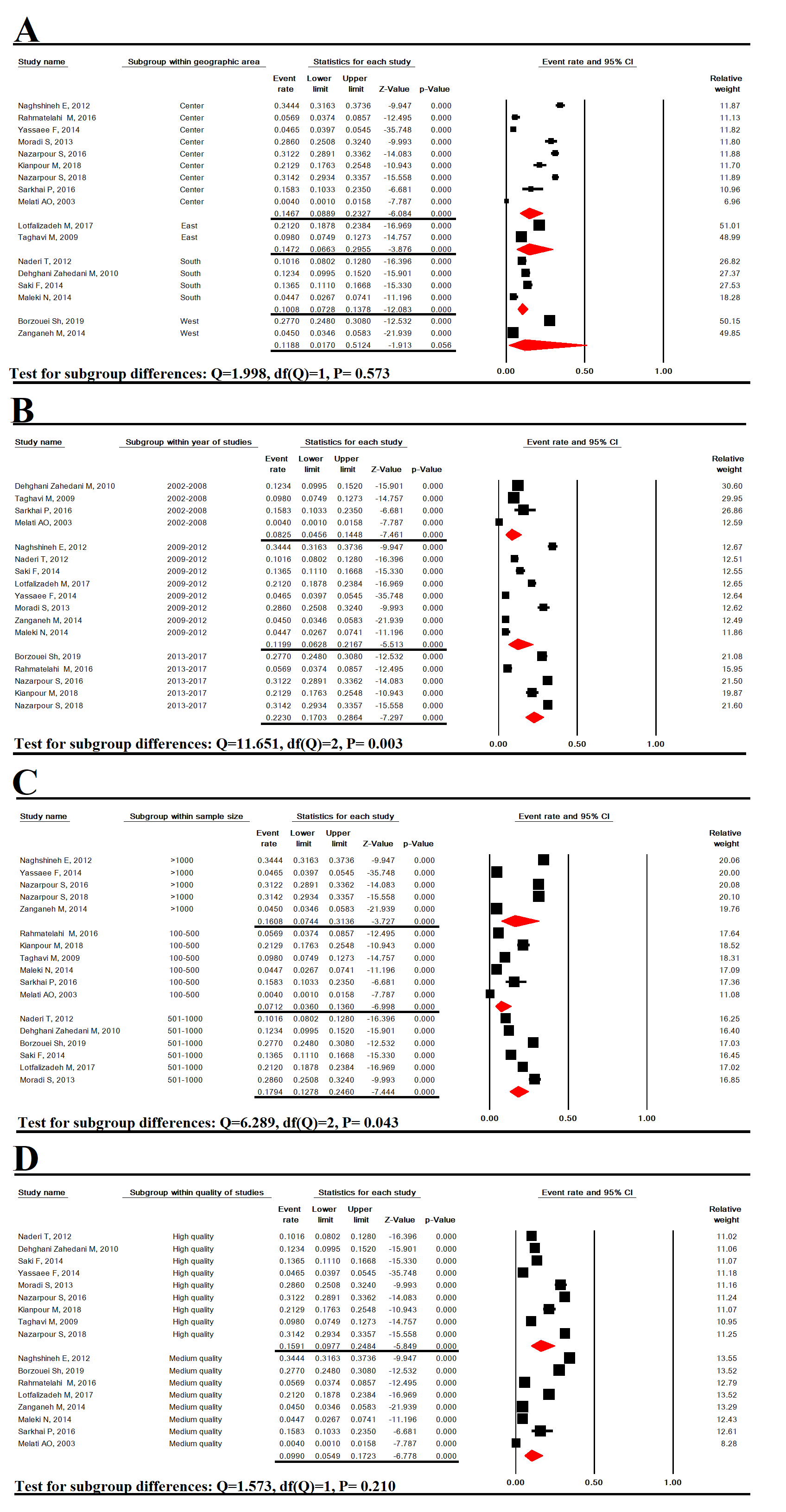

Supplement: Supplementary file 4 — Additional file 4. Subgroup analysis of hypothyroidism in pregnant Iranian women based on geographic regions (A), year of studies (B), sample size (C), and quality of studies (D) [file 12884_2020_3040_MOESM4_ESM.tif]

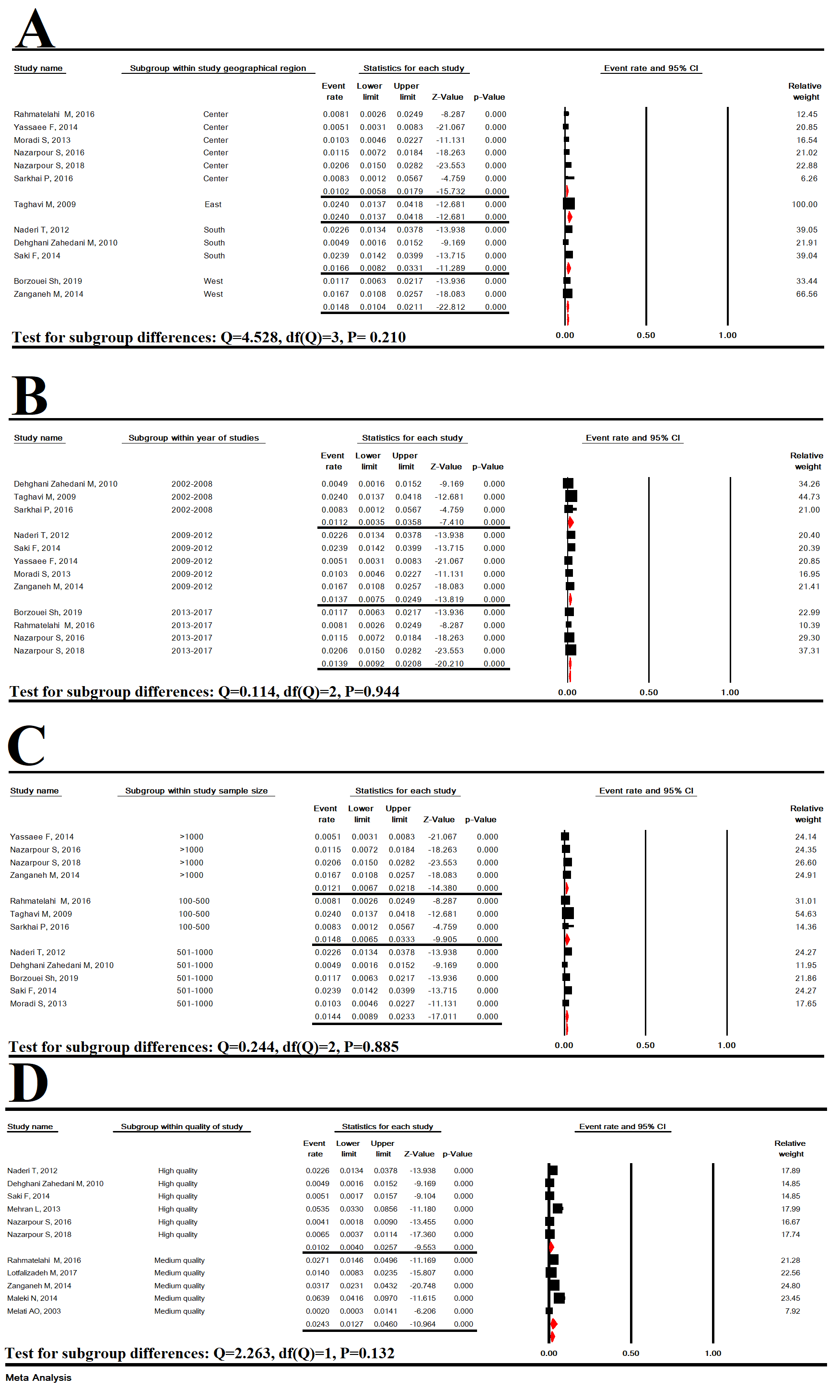

Supplement: Supplementary file 5 — Additional file 5. Subgroup analysis of clinical hypothyroidism in pregnant Iranian women based on geographic regions (A), year of studies (B), sample size (C), and quality of studies (D) [file 12884_2020_3040_MOESM5_ESM.tif]

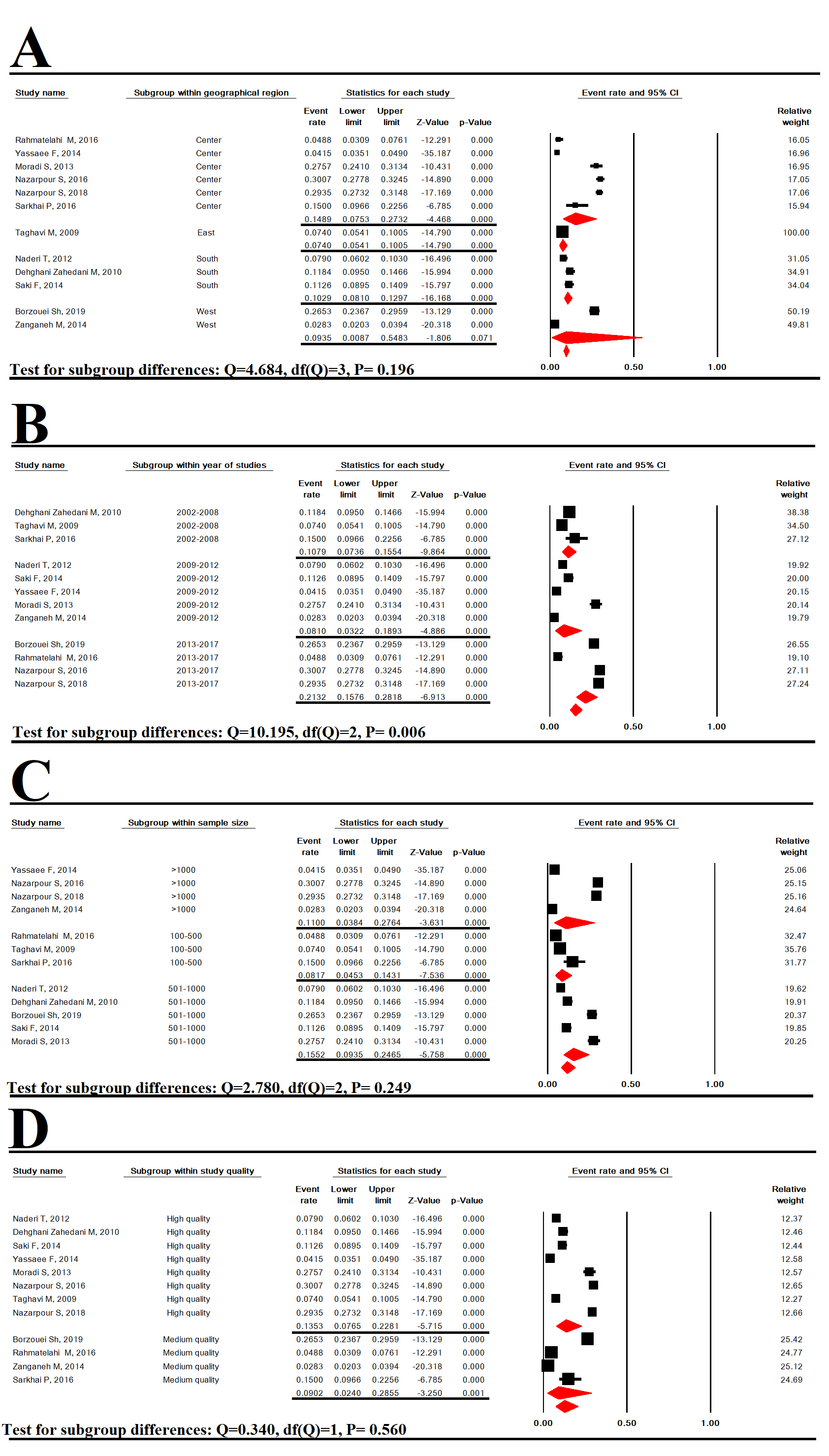

Supplement: Supplementary file 6 — Additional file 6. Subgroup analysis of subclinical hypothyroidism in pregnant Iranian women based on geographic regions (A), year of studies (B), sample size (C), and quality of studies (D) [file 12884_2020_3040_MOESM6_ESM.tif]

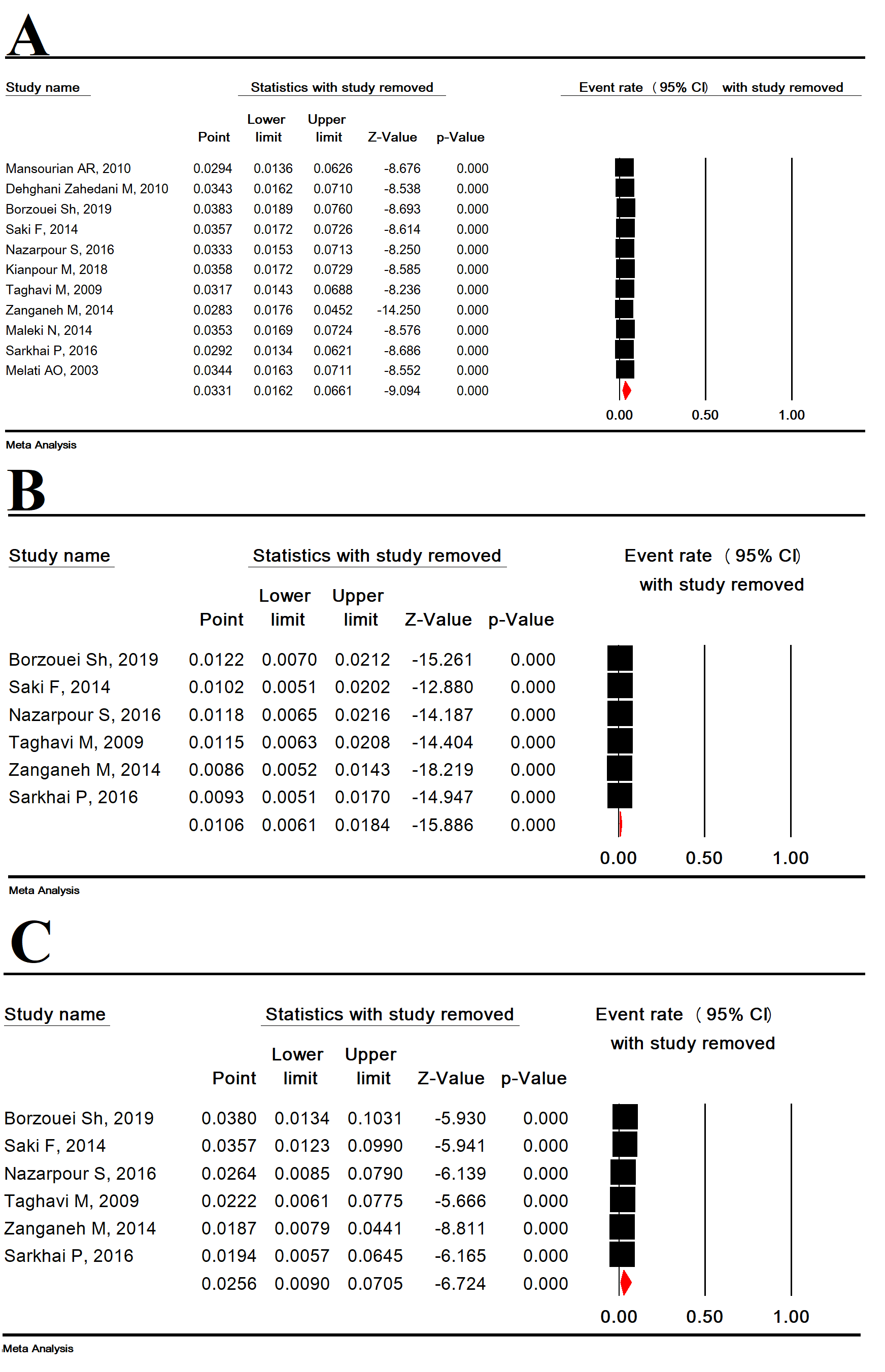

Supplement: Supplementary file 7 — Additional file 7. Sensitivity analysis for prevalence of hyperthyroidism (A), clinical hyperthyroidism (B), subclinical hyperthyroidism (C) in pregnant Iranian women [file 12884_2020_3040_MOESM7_ESM.tif]

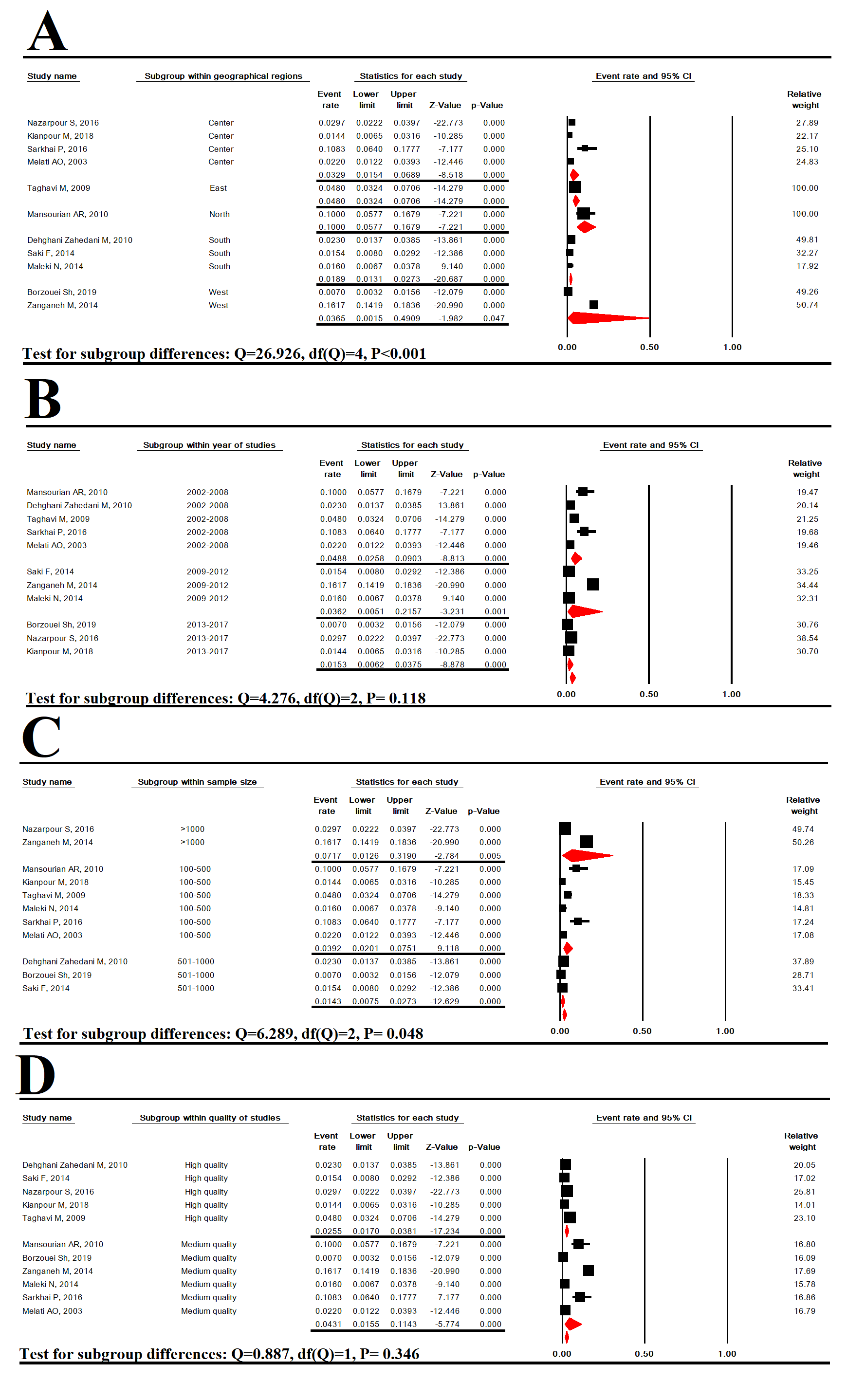

Supplement: Supplementary file 8 — Additional file 8. Subgroup analysis of hyperthyroidism in pregnant Iranian women based on geographic regions (A), year of studies (B), sample size (C), and quality of studies (D) [file 12884_2020_3040_MOESM8_ESM.tif]

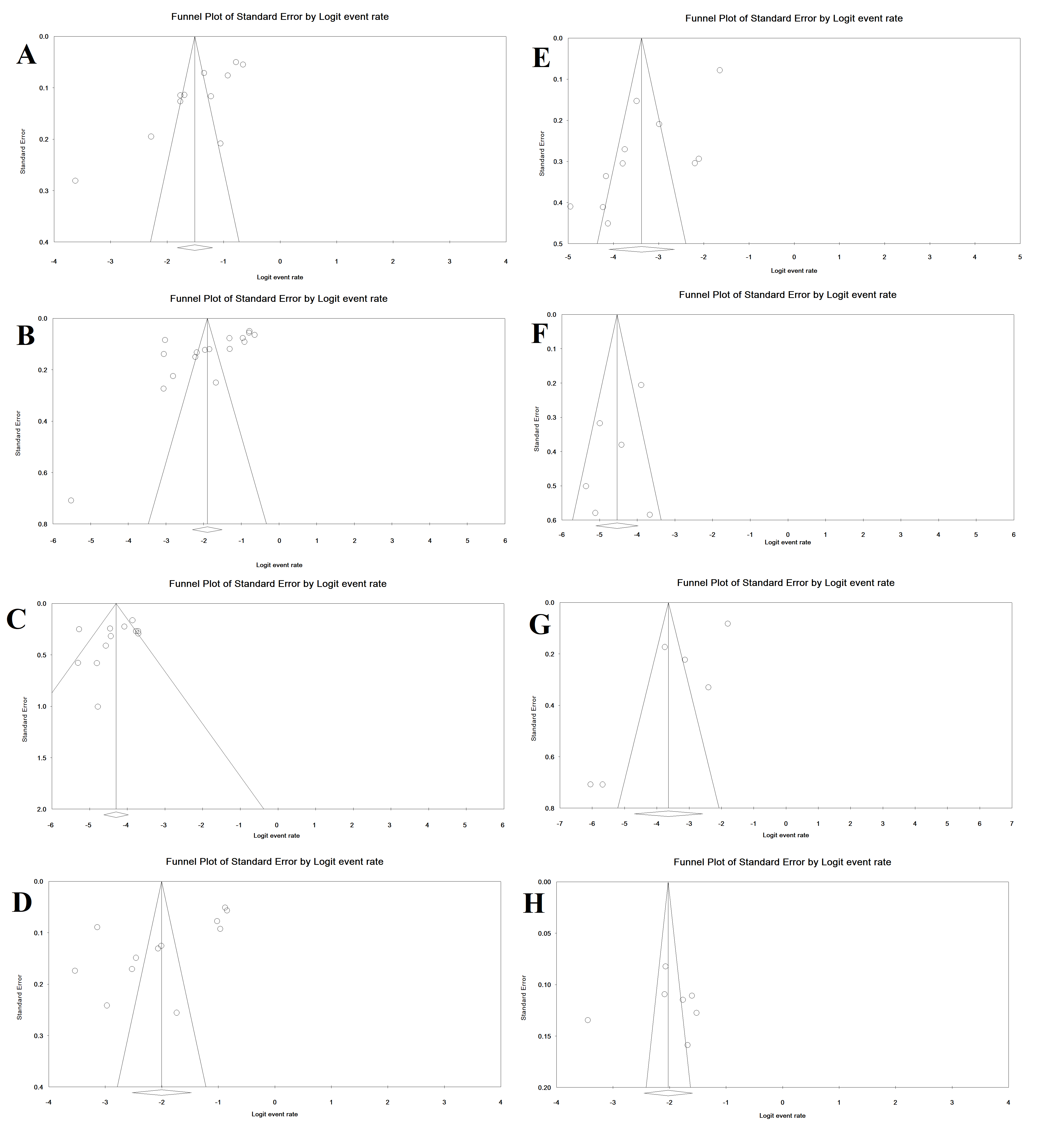

Supplement: Supplementary file 9 — Additional file 9. Funnel plot for thyroid function disorder (A), hypothyroidism (B), clinical hypothyroidism (C), subclinical hypothyroidism (D), hyperthyroidism (E), clinical hyperthyroidism (F), subclinical hyperthyroidism (G) and anti TPO (H) in pregnant Iranian women [file 12884_2020_3040_MOESM9_ESM.tif]
